# Garadacimab zur Langzeitprophylaxe bei hereditärem Angioödem

**DOI:** 10.1111/ddg.70276x

**Published:** 2026-08-04

**Authors:** Emel Aygören‐Pürsün, Regina Treudler, Petra Staubach, Inmaculada Martinez Saguer, Thomas Linhoff, Markus Magerl

**Affiliations:** ^1^ Goethe‐Universität Frankfurt Klinik für Kinder‐ und Jugendmedizin Frankfurt Deutschland; ^2^ Institut für Allergologie, Charité – Universitätsmedizin Berlin Mitglied der Freien Universität Berlin und der Humboldt‐Universität zu Berlin Berlin Deutschland; ^3^ Hautklinik und Poliklinik Universitätsmedizin Mainz Mainz Deutschland; ^4^ HZRM Hämophilie Zentrum Rhein‐Main Frankfurt Deutschland; ^5^ CSL Behring GmbH Hattersheim Deutschland; ^6^ Institut für Allergologie, Charité – Universitätsmedizin Berlin Mitglied der Freien Universität Berlin und der Humboldt‐Universität zu Berlin, Berlin und Fraunhofer‐Institut für Translationale Medizin und Pharmakologie ITMP, Allergologie und Immunologie Berlin Deutschland

**Keywords:** C1INH, Faktor XII, FXII, Garadacimab, HAE, Hereditäres Angioödem, Langzeitprophylaxe, LTP, VANGUARD, C1INH, factor XII, FXII, Garadacimab, HAE, hereditary angioedema, long‐term prophylaxis, LTP, VANGUARD

## Abstract

Das hereditäre Angioödem (*Hereditary angioedema*, HAE), eine seltene und belastende Erkrankung, die durch rezidivierende und spontane Gewebeschwellungsattacken gekennzeichnet ist, weist einen hohen ungedeckten therapeutischen Bedarf auf, da Patienten unter aktuellen prophylaktischen Behandlungen eine unzureichende Krankheitskontrolle erfahren. Das Ziel der HAE‐Behandlung gemäß den Leitlinien ist das Erreichen einer vollständigen Krankheitskontrolle und die Normalisierung des Lebens der Patienten. Faktor XII (FXII), der Hauptinitiator des Kontaktsystems, reguliert letztlich das Kallikrein‐Kinin‐System. Der aktivierte Faktor XII (FXIIa) wandelt Plasma‐Präkallikrein in Plasma‐Kallikrein um, welches das hochmolekulare Kininogen spaltet, um das vasoaktive Peptid Bradykinin zu erzeugen. In der Pathophysiologie des HAE führt eine Dysregulation des Kallikrein‐Kinin‐Systems zu einer übermäßigen Bradykininproduktion. Der monoklonale Anti‐FXIIa‐Antikörper Garadacimab ist zur Langzeitprophylaxe von HAE‐Attacken zugelassen. In der zulassungsrelevanten Phase‐III‐Studie (VANGUARD) und den laufenden offenen Phase‐III‐Erweiterungsstudien zeigte Garadacimab bei subkutaner Verabreichung (Aufdosierung mit 2 × 200 mg, gefolgt von 200 mg einmal monatlich) eine dauerhafte Wirksamkeit mit frühem Schutzbeginn und ein günstiges Langzeit‐Sicherheitsprofil. Basierend auf diesen und früheren klinischen Daten hat Garadacimab das Potenzial, Patienten den leitliniengemäßen Zielen der Krankheitskontrolle und Normalisierung des Lebens näherzubringen. Hier geben wir einen Überblick über die Ergebnisse des klinischen Entwicklungsprogramms von Garadacimab.

## EINFÜHRUNG

Das hereditäre Angioödem (HAE) ist eine seltene, potenziell lebensbedrohliche Erkrankung, die durch wiederkehrende und spontane Attacken entstellender und schmerzhafter Gewebeschwellungen gekennzeichnet ist, welche hauptsächlich die Haut, das Abdomen und die oberen Atemwege betreffen.^1^, ^2^ Neben der körperlichen Beeinträchtigung erfahren die Patienten eine verminderte gesundheitsbezogene Lebensqualität (HRQoL), da die Attacken Alltagsaktivitäten, die Arbeit und die psychische Gesundheit beeinträchtigen und letztlich Lebensstiländerungen erfordern.^3^, ^4^, ^5^

**ABBILDUNG 1:**
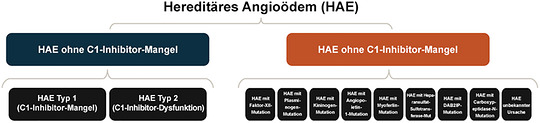
Die Rolle des Kallikrein‐Kinin‐Systems in der Pathophysiologie des HAE.^2^, ^6^, ^8^, ^15^, ^17^, ^70^, ^71^ *Abk*.: ANGPT, Angiopoietin‐1; C1INH, C1‐Esterase‐Inhibitor; CPN, Carboxypeptidase N; DAB2IP, „Disabled Homolog 2‐Interacting Protein“; FXII, Faktor XII; HAE, hereditäres Angioödem; HAE‐C1INH, hereditäres Angioödem mit C1‐Esterase‐Inhibitor‐Mangel oder ‐Dysfunktion; HAE‐nC1INH, hereditäres Angioödem mit normalem C1‐Esterase‐Inhibitor‐Spiegel; HSST, Heparansulfat‐Glucosamin‐3‐O‐Sulfotransferase 6; KNG, Kininogen 1; MYOF, Myoferlin; PLG, Plasminogen; UNK, unbekannter Ursprung

Das HAE ist eine autosomal‐dominant vererbte genetische Störung mit genetischer/klinischer Heterogenität; die HAE‐Typen werden nach dem Spiegel und der funktionellen Aktivität des C1‐Esterase‐Inhibitors (C1‐INH) (Abbildung 1) klassifiziert.^2^, ^6^

Das hereditäre Angioödem mit C1‐Esterase‐Inhibitor‐Mangel oder ‐Dysfunktion (HAE‐C1INH), der häufigste Typ, wird durch *SERPING1*‐Genvarianten verursacht, die entweder zu einem Mangel (HAE Typ 1) oder einer Dysfunktion (HAE Typ 2) von C1‐INH führen, was in der Folge zu einer Dysregulation des Kallikrein‐Kinin‐Systems und einer Überproduktion von Bradykinin führt.^2^, ^6^, ^7^, ^8^ HAE mit normalem C1‐INH‐Spiegel (HAE‐nC1INH) ist ein seltenerer Typ des HAE, der durch Genmutationen in anderen Proteinen verursacht wird, die an der Bildung oder Regulation von Bradykinin beteiligt sind.^2^, ^9^, ^10^

**ABBILDUNG 2:**
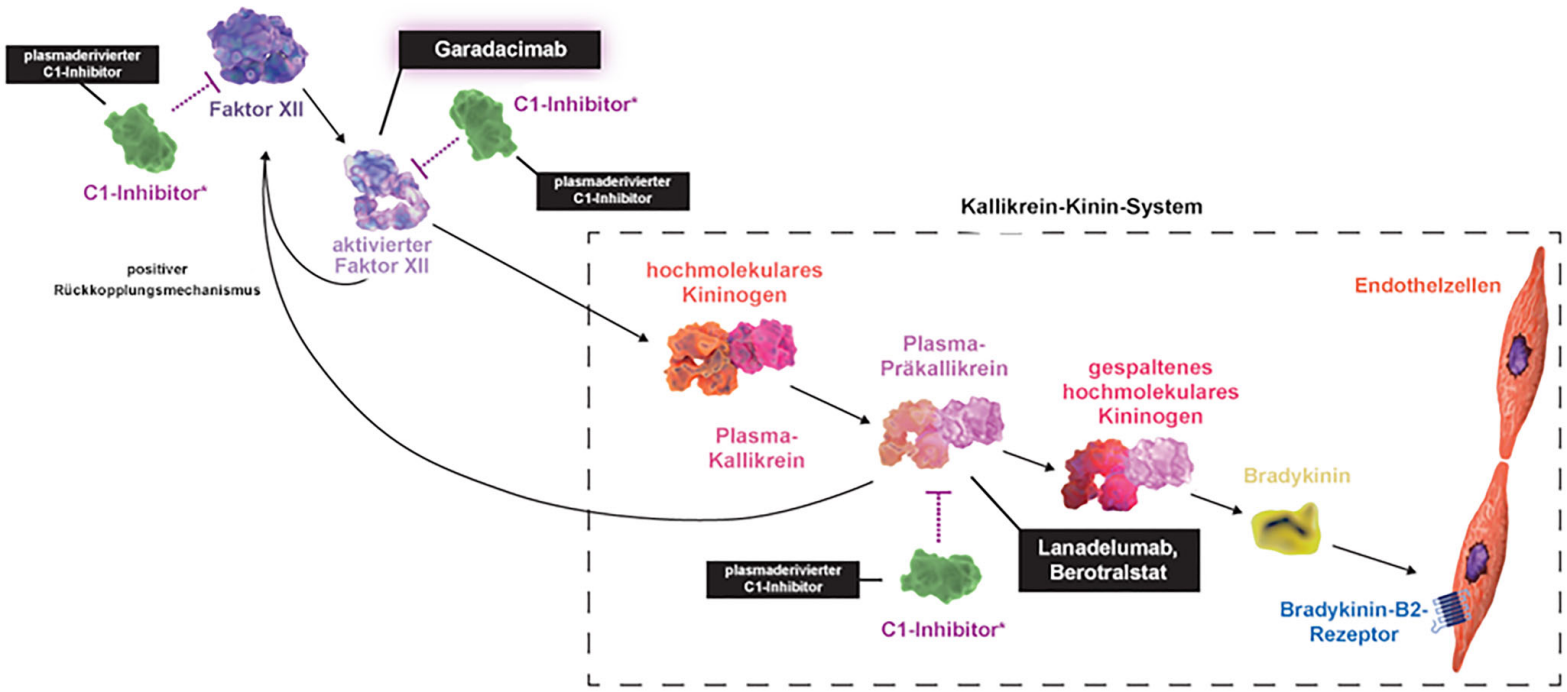
Darstellung des Kallikrein‐Kinin‐Systems.^11^, ^12^, ^27^ *Intrinsischer C1INH unter normalen physiologischen Bedingungen. *Abk*.: C1INH, C1‐Esterase‐Inhibitor; FXII, Faktor XII; FXIIa, aktivierter Faktor XII; pdC1INH, Plasma‐basierter C1‐Esterase‐Inhibitor.

Faktor XII ist der Hauptinitiator des Kontaktsystems und reguliert letztlich das Kallikrein‐Kinin‐System (Abbildung 2).^6^, ^7^, ^11^, ^12^ Das Kallikrein‐Kinin‐System ist eine proteolytische Kaskade, die zur Produktion der beiden proinflammatorischen Mediatoren führt, die für die Pathophysiologie des Angioödems verantwortlich sind: Plasma‐Kallikrein und Bradykinin.^12^, ^13^, ^14^ Die Aktivierung des Kallikrein‐Kinin‐Systems wird durch einen Prozess eingeleitet, der als „Kontaktaktivierung“ bezeichnet wird, wobei FXII bei der Bindung (Kontakt) an negativ geladene Oberflächen eine Konformationsänderung erfährt, was zur Bildung der aktivierten Protease FXIIa führt.^12^, ^14^ FXIIa wandelt Plasma‐Präkallikrein in seine aktive Form, das Plasma‐Kallikrein, um, welches das hochmolekulare Kininogen spaltet, um Bradykinin zu produzieren.^7^, ^12^ Nach Bindung an den Bradykinin‐Rezeptor B2 vermittelt Bradykinin vorübergehend die endotheliale Permeabilität, was zu vaskulärer Leckage und dem Ausstrom von Wasser, Ionen und Proteinen führt.^7^, ^12^, ^14^, ^15^ Zusammen bilden FXIIa und Plasma‐Kallikrein eine positive Rückkopplungsschleife, indem sie FXII spalten, weiteres FXIIa erzeugen und die Bradykininproduktion verstärken.^12^, ^14^

Unter normalen physiologischen Bedingungen reguliert C1INH (ein potenter Serinprotease‐Inhibitor) die Bradykininproduktion im Kallikrein‐Kinin‐System eng, indem es FXIIa und Plasma‐Kallikrein hemmt und die positive Rückkopplungsschleife unterbricht.^7^, ^16^ Bei Patienten mit HAE‐C1INH ist dieses System jedoch dysreguliert, was zu einer Überproduktion von Bradykinin führt.^12^, ^17^ Dies resultiert in einer übermäßigen endothelialen Permeabilität und den mit einer HAE‐Attacke verbundenen Schwellungen.^12^, ^15^

## AKTUELLE HAE‐LEITLINIE

Die von der *World Allergy Organization* (WAO)/*European Academy of Allergy and Clinical Immunology* (EAACI) und dem *Medical Advisory Board* der *United States Hereditary Angioedema Association* (US HAEA) empfohlenen Ziele bei der HAE‐Behandlung sind die vollständige Krankheitskontrolle und die Normalisierung des Alltags der Patienten, indem die Beeinträchtigung von Alltagsaktivitäten reduziert oder eliminiert wird.^2^, ^6^ Keine HAE‐Attacken mehr zu erleben, ist eine wichtige Voraussetzung für die vollständige Krankheitskontrolle.^2^ Die WAO/EAACI‐Leitlinie empfiehlt die Bedarfsbehandlung (*on‐demand*) zur Therapie von HAE‐Attacken, die Kurzzeitprophylaxe zur Minimierung des Attackenrisikos im Vorfeld auslösender Ereignisse wie chirurgischer oder zahnärztlicher Eingriffe sowie die Langzeitprophylaxe (LTP) zur Prävention von Attacken.^2^ Die WAO/EAACI‐Leitlinie stellt zudem fest, dass die LTP die einzige Behandlungsstrategie ist, mit der derzeit eine vollständige Krankheitskontrolle und Lebensnormalisierung erreicht werden kann, und dass der potenzielle Nutzen eines Wechsels zur LTP bei jeder klinischen Visite des Patienten evaluiert werden sollte.^2^ Die empfohlenen Erstlinien‐Behandlungen für die LTP (Tabelle 1) umfassen die C1INH‐Therapie in Form von plasmabasiertem C1INH (pdC1INH).^2^ Die C1INH‐Therapie soll mangelndes oder dysfunktionales C1INH ersetzen und dadurch die Kallikrein‐ und Bradykininproduktion regulieren (Abbildung 2).^2^ pdC1INH ist in den USA als Cinryze^®^ (intravenös [i.v.]) oder Haegarda^®^ (subkutan [s.c.]) und in Europa als Cinryze^®^ (i.v.) oder Berinert^®^ 2000/3000 (s.c.) als LTP zugelassen.^18^, ^19^, ^20^, ^21^ Weitere empfohlene Erstlinien‐LTP‐Optionen sind Lanadelumab (Takhzyro^®^; s.c.), ein vollhumaner monoklonaler Antikörper gegen Plasma‐Kallikrein, und Berotralstat (Orladeyo^®^; oral), ein selektiver Inhibitor von Plasma‐Kallikrein (Abbildung 2).^2^, ^22^, ^23^ Seit der Veröffentlichung der aktuellen Behandlungsleitlinie wurden zwei zusätzliche Therapieoptionen für die LTP gegen HAE zugelassen: Garadacimab (Andembry^®^; s.c.; unten beschrieben) und Donidalorsen (Dawnzera^®^; s.c.), ein gegen Plasma‐Präkallikrein gerichtetes Antisense‐Oligonukleotid.^24^, ^25^, ^26^

**TABELLE 1:** Zugelassene Erstlinien‐Behandlungen, die von aktuellen Behandlungsleitlinien für die Langzeitprophylaxe (LTP) bei HAE empfohlen werden.^2^, ^6^

Behandlung	Wirkmechanismus	Hauptergebnisse aus klinischen Studien	Zugelassene Indikation (FDA/EMA), Dosierungsschema und Art der Anwendung
pdC1INH (i.v.; Cinryze^®^, Takeda)^6^, ^18^, ^20^, ^63^, ^64^	Hemmt Plasma‐Kallikrein, FXIIa, FXIa, C1s, C1r, MASP‐1, MASP‐2 und Plasmin	Primärer Endpunkt Mittlere monatliche Attackenrate: ∼2,09^*^ Reduktion der mittl. monatl. Attackenrate: n. b. (NR)*Sekundärer Endpunkt* Mittl. monatl. ODT‐Injektionen: 1,57^†^	Patienten ab 6 Jahren mit HAE (FDA/EMA): 500 IE (6–11 J.) oder 1000 IE (≥ 12 J.) i.v. alle 3–4 Tage. Die Dosis kann basierend auf dem individuellen Ansprechen angepasst werden
pdC1INH (s.c.; Berinert^®^ 2000/3000, Haegarda^®^, CSL Behring)^6^, ^19^, ^21^, ^64^, ^65^	Hemmt Plasma‐Kallikrein, FXIIa, FXIa, C1s, C1r, MASP‐1, MASP‐2 und Plasmin	Primärer Endpunkt Mittlere monatliche Attackenrate: 0,52 Reduktion der mittl. monatl. Attackenrate^‡^: 84 % Sekundärer Endpunkt Anteil attackenfreier Patienten: 40 %	Patienten ab 6 Jahren mit HAE (FDA); Erwachsene und jugendliche Patienten mit HAE‐C1INH (EMA): 60 IE/kg s.c. alle 3–4 Tage
Lanadelumab (Takhzyro^®^, Takeda)^22^, ^66^, ^67^	Hemmt Plasma‐Kallikrein	Primärer Endpunkt Mittlere monatliche Attackenrate: 0,26 (q2w), 0,53 (q4w) Reduktion der mittl. monatl. Attackenrate^‡^: 87 % (q2w), 73 % (q4w) Sekundärer Endpunkt Anteil attackenfreier Patienten: 44 % (q2w), 31 % (q4w)	Patienten ab 12 Jahren mit HAE (FDA/EMA): 300 mg s.c. q2w / 300 mg q4w^¶^ Patienten von 2 bis < 12 Jahren (nur EMA): 150 mg q4w (10 bis < 20 kg)^**^ oder 150 mg q2w (20 bis < 40 kg)^††^ oder 300 mg q2w (≥ 40 kg)^‡‡^ Patienten von 2 bis < 12 Jahren (nur FDA): 150 mg q4w (≥ 2 bis < 6 Jahre) 150 mg q2w (≥ 6 bis < 12 Jahre)^§§^
Berotralstat (Orladeyo^®^, BioCryst)^23^, ^68^, ^69^	Hemmt Plasma‐Kallikrein	Primärer Endpunkt Monatl. Attackenrate: 1,31 (150 mg), 1,65 (110 mg) Reduktion der monatl. Attackenrate^‡^: 44 % (150 mg), 30 % (110 mg) Sekundärer Endpunkt Anteil der Pat. mit einer ≥ 90 % Reduktion der Attackenrate: ≤ 23 %^§^	Patienten ab 12 Jahren mit HAE (FDA/EMA): 150 mg qd oral

*Abk*.: C1r, Komplementkomponente 1r; C1s, Komplementkomponente 1s; EMA, Europäische Arzneimittel‐Agentur; FDA, Food and Drug Administration; FXIa, aktivierter Faktor XI; FXIIa, aktivierter Faktor XII; HAE, hereditäres Angioödem; HAE‐C1INH, HAE durch C1INH‐Mangel oder ‐dysfunktion; IE (IU), Internationale Einheiten; i.v., intravenös; LTP, Langzeitprophylaxe; MASP, Mannose‐bindende Lektin‐assoziierte Serinprotease; n. b. (NR), nicht berichtet; ODT, Bedarfstherapie (*On‐demand*‐Therapie); pdC1INH, Plasma‐basierter C1 Inhibitor; q2w, alle 2 Wochen; q4w, alle 4 Wochen; qd, einmal täglich; s.c., subkutan

* Berechnet aus dem Mittelwert von 6,26 Attacken während des 12‐wöchigen Behandlungszeitraums

^†^ Berechnet aus dem Mittelwert von 4,7 Notfallinfusionen (rescue infusions) während eines 12‐wöchigen Behandlungszeitraums

^‡^ Reduktion gegenüber Placebo

^§^ Nicht signifikant verschieden von Placebo

^¶^ Kann in Betracht gezogen werden, wenn die Krankheit über > 6 Monate gut kontrolliert ist (zum Beispiel Patient ist attackenfrei)

** Dosis kann auf 150 mg q3w erhöht werden, wenn die Kontrolle unzureichend ist

^††^ Dosis kann auf 150 mg q4w reduziert werden, wenn stabil

^‡‡^ Dosis kann auf 300 mg q4w reduziert werden, wenn stabil

^§§^ Dosis kann auf 150 mg q4w reduziert werden, wenn stabil.

## GARADACIMAB

### Wirkmechanismus

Die gezielte Hemmung von FXIIa, dem Hauptinitiator des Kallikrein‐Kinin‐Systems, ist eine innovative Behandlungsstrategie für HAE.^16^, ^27^ Garadacimab ist ein vollhumaner, Immunglobulin‐G4‐Anti‐FXIIa‐monoklonaler *First‐in‐Class*‐Antikörper, der FXIIa mit hoher Affinität, Potenz und Spezifität hemmt und somit den Beginn des Bradykinin‐bildenden Kallikrein‐Kinin‐Systems blockiert (Abbildung 2).^27^, ^28^, ^29^, ^30^

### Klinisches Entwicklungsprogramm und Zulassungsübersicht

Garadacimab wird weiterhin in einem umfassenden klinischen Programm bei erwachsenen (Alter > 17 Jahre), jugendlichen (Alter 12–17 Jahre) und pädiatrischen (Alter 2–11 Jahre) Patienten mit HAE zur LTP untersucht (Tabelle 2).^28^, ^31^, ^32^, ^33^, ^34^ Basierend auf den Ergebnissen, die mit Garadacimab s.c. (2 × 200 mg Aufdosierung, gefolgt von 200 mg einmal monatlich) in der zulassungsrelevanten Phase‐III‐Studie (VANGUARD) beobachtet wurden, hat Garadacimab die Zulassung durch die *Europäische Arzneimittel‐Agentur* (EMA), die US‐amerikanische *Food and Drug Administration* (FDA) sowie Behörden in Australien, Kanada, Japan, der Schweiz, den VAE und Großbritannien als LTP zur Prävention von HAE‐Attacken bei Erwachsenen und Jugendlichen ab 12 Jahren erhalten.^24^, ^25^, ^35^, ^36^, ^37^, ^38^, ^39^, ^40^, ^41^

**TABELLE 2:** Übersicht über die klinischen Studien zu Garadacimab.

	Phase II^28^, ^31^	Zulassungsrelevante Phase III (VANGUARD)^32^	Phase III OLE^†^, ^33^	Pädiatrische Phase‐III‐Studie^†^, ^34^
Studiendesign	Multizentrische, doppelblinde, placebokontrollierte RCT mit OLE	Doppelblinde, placebokontrollierte RCT	Offene Erweiterungsstudie	Multizentrisch, offen, einarmig
Geeignete Patienten	HAE‐C1INH HAE‐FXII/‐PLG	HAE‐C1INH	*Rollover*‐Patienten aus Phase‐II‐ oder Phase‐III‐Studien sowie neu rekrutierte Patienten	HAE‐C1INH
Anzahl der Patienten	44^*^	64	161	20 (Zielgröße)
Alter (Jahre)	18–65	≥ 12	≥ 12	2–11
Behandlungsarme	TP1: i.v.‐Initialdosis (*Loading Dose*) Garadacimab 40 mg/100 mg/300 mg oder Placebo an Tag 1, gefolgt von Garadacimab 75 mg/200 mg/600 mg oder Placebo q1m s.c.	s.c.‐Initialdosis Garadacimab (2 × 200 mg Dosen) oder volumenentsprechendes Placebo an Tag 1, gefolgt von Garadacimab 200 mg oder Placebo q1m s.c.	Garadacimab 200 mg q1m s.c. (neu rekrutierte Patienten erhielten 2 × 200 mg s.c. Initialdosis als erste Dosis)	Dosis A s.c. (Alter 2–5 Jahre) Dosis B s.c. (Alter 6–11 Jahre)
	TP2: Garadacimab 200 mg/600 mg q1m s.c.			
Studiendauer	TP1: 13 Wochen TP2: ≥ 44 Wochen	6 Monate	≥ 12 Monate	12 Monate
Primärer Endpunkt	Zeitnormalisierte Anzahl der HAE‐Attacken	Zeitnormalisierte Anzahl der HAE‐Attacken	TEAE	TEAE, PK

*Abk*.: HAE, hereditäres Angioödem; HAE‐C1INH, HAE mit C1‐Esterase‐Inhibitor‐Mangel oder ‐Dysfunktion; HAE‐FXII/‐PLG, HAE mit Faktor XII‐ oder Plasminogen‐Mutationen; i.v., intravenös; OLE, offene Erweiterungsstudie; PK, Pharmakokinetik; q1m, jeden Monat; RCT, randomisierte kontrollierte Studie; s.c., subkutan; TEAE, therapiebedingtes unerwünschtes Ereignis; TP, Behandlungszeitraum

* Einschließlich sechs Patienten mit HAE‐XII/‐PLG; Details zu diesen Patienten werden separat berichtet;^51^

^†^ Laufende Studie.

Die Wirksamkeit und Sicherheit von Garadacimab zur LTP gegen HAE‐Attacken wurde zunächst in einer randomisierten Phase‐II‐Dosisfindungsstudie über zwei Behandlungszeiträume von 13 Wochen und ≥ 44 Wochen untersucht (NCT03712228; Tabelle 2).^28^, ^31^

Im Anschluss an die Phase‐II‐Dosisfindungsstudie wurde Garadacimab 200 mg s.c. für die weitere Untersuchung in einer doppelblinden, randomisierten, placebokontrollierten, zulassungsrelevanten Phase‐III‐Studie (VANGUARD) ausgewählt (NCT04656418) (Tabelle 2).^32^ In dieser Studie wurden 64 erwachsene und jugendliche Patienten mit HAE‐C1INH im Verhältnis 3:2 entweder auf einmal monatlich Garadacimab 200 mg s.c. (n = 39) oder Placebo (n = 25) über eine Behandlungsdauer von 6 Monaten randomisiert. Der primäre Endpunkt war die zeitnormalisierte Anzahl der HAE‐Attacken (monatliche Attackenrate), und die sekundären Sicherheitsendpunkte waren unerwünschte Ereignisse (*adverse events*; AE), einschließlich AE von besonderem Interesse (*adverse events of special interest*; AESI) gemäß Protokoll (abnorme Blutungsereignisse, thromboembolische Ereignisse, schwere Überempfindlichkeit und Anaphylaxie), schwerwiegende unerwünschte Ereignisse (*serious adverse events*; SAE) sowie klinisch signifikante Anomalien in Laboruntersuchungen; zudem wurden Konzentrationen von Anti‐Drug‐Antikörpern gemessen.^32^

Die langfristige (≥ 12 Monate) Wirksamkeit und Sicherheit von Garadacimab 200 mg s.c. einmal monatlich bei erwachsenen und jugendlichen Patienten wird derzeit in einer laufenden offenen Phase‐III‐Erweiterungsstudie (Open‐Label Extension; OLE) untersucht (NCT04739059) (Tabelle 2).^33^ Insgesamt nahmen 161 Patienten an der OLE teil: 35 aus dem Behandlungszeitraum 2 (TP2) der Phase‐II‐Studie, 57 aus der zulassungsrelevanten Phase‐III‐Studie (VANGUARD) und 69 neu eingeschlossene Patienten.^33^ Der primäre Endpunkt der Phase‐III‐OLE‐Studie ist das Auftreten behandlungsbedingter unerwünschter Ereignisse (*treatment‐emergent adverse events*; TEAE) bei Patienten mit HAE‐C1INH. Sekundäre Endpunkte umfassen Langzeit‐Wirksamkeitsparameter (zum Beispiel monatliche Attackenrate, Reduktion der Attackenrate gegenüber dem *Run‐in*, Anzahl moderater und schwerer Attacken) und Sicherheitsparameter (zum Beispiel SAE, Todesfälle, TEAE, die zum Abbruch führen, und AESI gemäß Protokoll).^33^

Garadacimab wird zudem über 12 Monate in einer laufenden multizentrischen, einarmigen, offenen Phase‐III‐Studie bei pädiatrischen Patienten (Alter 2–11 Jahre) mit HAE‐C1INH untersucht (NCT05819775) (Tabelle 2).^34^ Diese Studie hat ein Rekrutierungsziel von 20 Patienten; zu ihren primären Endpunkten gehören TEAE und Pharmakokinetik. Sekundäre Wirksamkeitsendpunkte umfassen die monatliche Attackenrate.^34^

### Pharmakokinetik und Pharmakodynamik von Garadacimab

Daten von Erwachsenen und Jugendlichen, die Garadacimab in klinischen Phase‐I‐, ‐II‐ und ‐III‐Studien an gesunden Probanden und HAE‐Patienten erhielten, wurden zur Entwicklung von Populations‐Pharmakokinetik‐ und ‐Pharmakodynamik‐Modellen herangezogen. Daten der Phase‐II‐ und ‐III‐Studienteilnehmer dienten zur Erstellung eines Expositions‐Wirkungs‐Modells, um die Begründung für die Dosierung von 200 mg Garadacimab einmal monatlich zu evaluieren.^42^

Modelle, welche die Pharmakokinetik und Pharmakodynamik von Garadacimab adäquat charakterisierten, zeigten, dass das *steady state* bereits eine Woche nach der ersten Verabreichung einer Initialdosis (2 × 200 mg s.c.) erreicht wurde, bei einer Eliminationshalbwertszeit von 8,5 Tagen.^42^ Die Expositions‐Wirkungs‐Analyse ergab, dass Garadacimab 200 mg s.c. einmal monatlich dazu führte, dass 75 % der Patienten den therapeutischen Zielschwellenwert erreichten (90 % Reduktion des relativen Attackenrisikos gegenüber dem *Run‐in*).^42^ Diese Daten unterstützen die Anwendung von Garadacimab 200 mg s.c. einmal monatlich ohne Notwendigkeit einer Dosisanpassung.^42^

### Wirksamkeit von Garadacimab

In der 6‐monatigen zulassungsrelevanten Phase‐III‐Studie (VANGUARD) war die monatliche Attackenrate (95 %‐Konfidenzintervalle [KI]) für Patienten in beiden Behandlungsarmen während des *Run‐ins* ähnlich (3,1 [2,4–3,7] für den Arm mit 200 mg s.c. Garadacimab einmal monatlich und 2,5 [2,1–2,9] für den Placeboarm).^32^ Die mittlere monatliche Attackenrate (95 %‐KI) war unter Garadacimab niedriger (0,27 [0,05–0,49]) als unter Placebo (2,01 [1,44–2,57]), was einer statistisch signifikanten mittleren Reduktion von 87 % entspricht (p < 0,0001) (Tabelle 3).^32^ Die mediane monatliche Attackenrate (Interquartilsabstand [IQR]) betrug 0 (0,0–0,31) unter Garadacimab gegenüber 1,35 (1,00–3,20) unter Placebo, wobei die prozentuale Reduktion gegenüber dem *Run‐in* nicht berichtet wurde. Zudem blieben 62 % der Patienten unter Garadacimab über den 6‐monatigen Studienzeitraum attackenfrei, im Vergleich zu keinem der Patienten unter Placebo (p < 0,0001).^32^

**TABELLE 3:** Wichtigste Wirksamkeitsdaten aus der zulassungsrelevanten Phase‐III‐ (VANGUARD) und der Phase‐III‐OLE‐Studie.

Parameter	Zulassungsrelevante Phase‐III‐Studie (VANGUARD)^32^		Phase III OLE‐Studie (n = 161)^33^	
	*Garadacimab 200 mg (n = 39)*	*Placebo (n = 25)*	*Garadacimab*	*Run‐in*
Behandlungszeitraum, Monate	6		Median (IQR):13,8 (11,9–16,3)	
Mittlere monatliche Attackenrate (95 %‐KI)	0,27 (0,05–0,49)	2,01 (1,44–2,57)	0,16 (0,10–0,22)	3,57 (3,20–3,95)
Mittlere prozentuale Differenz (95 %‐KI) der monatl. Attackenzahl vs. Placebo/*Run‐in*	−87 (–96 bis –58)		−95 (–97 bis –93)	
Mediane monatliche Attackenrate (IQR)	0 (0,00–0,31)	1,35 (1,00–3,20)	0 (0,00–0,13)	2,85 (1,90–4,35)
Mediane prozentuale Differenz (IQR) der Attackenzahl vs. *Run‐in*	–^*^		−100 (–100 bis –96)	
Anzahl (%) attackenfreier Patienten^†^	24 (62)	0	96 (60)	–
Mittl. monatl. Attackenrate mit Bedarf an ODT (95 %‐KI)	0,23 (0,02–0,45)	1,86 (1,26–2,46)	0,14 (0,09–0,20)	2,98 (2,57–3,40)
Mittl. monatl. Anzahl moderater/schwerer Attacken (95 %‐KI)	0,13 (0,03–0,22)	1,35 (0,86–1,84)	0,11 (0,07–0,15)	2,59 (2,26–2,92)

*Abk*.: KI, Konfidenzintervall; HAE, hereditäres Angioödem; ODT, Bedarfsbehandlung (*On‐demand*‐Therapie); OLE, offene Erweiterungsstudie (*Open‐label extension*).

* Die mediane prozentuale Differenz (IQR) der Attackenzahl gegenüber dem *Run‐in* wurde in der zulassungsrelevanten Phase‐III‐Studie (VANGUARD) nicht berichtet.^32^

^†^ Attackenfreie Patienten sind jene, die eine 100%ige Reduktion der HAE‐Attackenrate aufwiesen.

Die Ergebnisse der ersten Interimsanalyse der laufenden Phase‐III‐OLE‐Studie (Datenstichtag: Februar 2023) stimmten mit denen der 6‐monatigen zulassungsrelevanten Phase‐III‐Studie (VANGUARD) überein und zeigten eine dauerhafte Wirksamkeit sowie einen anhaltenden Schutz vor HAE‐Attacken unter Garadacimab 200 mg s.c. einmal monatlich bei einer medianen Exposition von 13,8 Monaten (Tabelle 3).^32^, ^33^ Die mittlere monatliche Attackenrate (95 %‐KI) betrug 0,16 (0,10–0,22) während der Garadacimab‐Behandlung gegenüber 3,57 (3,20–3,95) während des *Run‐ins*, was einer mittleren Reduktion von 95 % entspricht.^33^ Die mediane monatliche Attackenrate (IQR) lag bei 0 (0,0–0,13) unter Garadacimab gegenüber 2,85 (1,90–4,35) im *Run‐in*, entsprechend einer medianen Reduktion von 100 %.^33^ Konsistent mit früheren Daten waren 60 % der Patienten unter Garadacimab bis zum Datenschnitt attackenfrei.^33^ Zudem behielten 21/22 (95 %) der Patienten, die Garadacimab in der *Rollover*‐Gruppe erhielten und bereits während der gesamten VANGUARD‐Studie attackenfrei geblieben waren, diesen Status über die gesamte OLE hinweg bei (mediane Exposition von 14,0 Monaten).^33^

Ergebnisse einer *Post‐hoc*‐integrierten Wirksamkeitsanalyse, die die 6‐monatige Phase‐III‐Studie (VANGUARD) und die Langzeit‐OLE‐Studien (mediane Exposition: 1,2 Jahre) umfasst, bestätigten die dauerhafte Wirksamkeit und den anhaltenden Schutz von Garadacimab 200 mg s.c. einmal monatlich.^43^ Die mittlere monatliche Attackenrate betrug 0,17 (Standardabweichung 0,40) unter Garadacimab gegenüber 3,55 (2,40) im *Run‐in*, was einer mittleren Reduktion von 94 % entspricht.^43^

Garadacimab zeigt einen frühen Schutzbeginn gegen HAE‐Attacken, wie in einer Post‐hoc‐Analyse der VANGUARD‐Studie ermittelt wurde. Bereits eine Woche nach der ersten Verabreichung reduzierte Garadacimab die mittlere monatliche Attackenrate gegenüber Placebo.^44^ In Woche 1 betrug die mittlere monatliche Attackenrate (95 %‐KI) in der Garadacimab‐Gruppe 0,11 (–0,11 bis 0,34) gegenüber 3,07 (2,41–3,73) im *Run‐in*, verglichen mit 1,81 (0,74–2,88) gegenüber 2,52 (2,13–2,91) im *Run‐in* der Placebogruppe.^44^ Das Erreichen der *Steady‐State*‐Exposition nach der ersten Initialdosis könnte dem beobachteten frühen Schutzbeginn zugrunde liegen.^42^ Die mittlere monatliche Anfallsrate bis zum 6. Monat blieb unter Garadacimab niedriger als im *Run‐in* und unter Placebo.^44^

Darüber hinaus war Garadacimab in einer indirekten Vergleichsstudie mit Adjustierung (*Matching‐adjusted Indirect Comparison*) mit statistisch signifikanten Verbesserungen bei allen untersuchten Wirksamkeitsergebnissen gegenüber Lanadelumab 300 mg (alle 4 Wochen) assoziiert. Daten aus der Phase‐II‐ und der zulassungsrelevanten Phase‐III‐Studie von Garadacimab wurden mit publizierten Phase‐III‐Daten der Lanadelumab HELP‐Studie (NCT02586805) verglichen. Garadacimab 200 mg s.c. einmal monatlich reduzierte die Gesamt‐HAE‐Attackenrate (Rate Ratio [RR] 0,29 [95 %‐KI 0,13–0,63]), die Rate der Attacken, die eine Bedarfsbehandlung erforderten (RR 0,29 [95 %‐KI 0,13–0,66]), die Rate moderater und/oder schwerer HAE‐Attacken (RR 0,15 [95 %‐KI 0,05–0,49]) und erhöhte den Anteil attackenfreier Patienten (Hazard Ratio 3,25 [95 %‐KI 1,45–7,29]) gegenüber Lanadelumab 300 mg alle 4 Wochen (p < 0,05 für jeden Parameter).^45^ Garadacimab reduzierte zudem signifikant die Rate moderater und/oder schwerer HAE‐Attacken (RR 0,25 [95 %‐KI 0,07–0,84]; p < 0,05) gegenüber Lanadelumab 300 mg alle 2 Wochen, reduzierte jedoch die Gesamt‐HAE‐Attackenrate nicht signifikant.^45^ Konsistent mit diesem Ergebnis reduzierte Garadacimab 200 mg einmal monatlich in einer Netzwerk‐Metaanalyse (NMA) die HAE‐Attackenrate gegenüber C1INH s.c., Berotralstat und Lanadelumab 300 mg alle 4 Wochen, jedoch nicht signifikant gegenüber Lanadelumab 300 mg alle 2 Wochen.^45^ Diese NMA kam zu dem Schluss, dass Garadacimab die höchste Wahrscheinlichkeit aufwies, die effektivste Behandlung zur Reduktion von HAE‐Attacken zu sein (73 %), wobei Lanadelumab alle 2 Wochen den zweiten Platz belegte (26 %).^45^ Garadacimab war durchweg mit der höchsten Wahrscheinlichkeit assoziiert, Wirksamkeitsendpunkte zu erreichen, einschließlich eines erhöhten Anteils attackenfreier Patienten (55 % gegenüber 28 % bei Lanadelumab alle 2 Wochen), einer Reduktion von HAE‐Attacken, die eine Bedarfsbehandlung erfordern (53 % gegenüber 35 % bei C1INH s.c.), und einer Reduktion moderater und/oder schwerer HAE‐Attacken (98 % gegenüber 1 % bei C1INH s.c.).^45^

### Sicherheit von Garadacimab

Während der 6‐monatigen zulassungsrelevanten Phase‐III‐Studie (VANGUARD) war der Anteil der Patienten, bei denen mindestens ein TEAE auftrat, in beiden Behandlungsarmen vergleichbar (64 % der mit Garadacimab behandelten Patienten [insgesamt 75 TEAE] gegenüber 60 % der Patienten in der Placebogruppe [insgesamt 54 TEAE]).^32^ Auch der Anteil der TEAE, die als mit der Studienbehandlung im Zusammenhang stehend eingestuft wurden, war zwischen dem Garadacimab‐Arm (10 %) und dem Placebo‐Arm (12 %) vergleichbar (Tabelle 4).^32^ Die Mehrheit der TEAE klang in beiden Behandlungsgruppen vor Ende der Studie ab.^32^

**TABELLE 4:** Wichtigste Sicherheitsdaten aus der zulassungsrelevanten Phase‐III‐ (VANGUARD) und der Phase‐III‐OLE‐Studie.

Endpunkt, n (%)	Zulassungsrelevante Phase‐III‐Studie (VANGUARD)^32^		Phase III OLE‐Studie (n = 161)^33^
	*Garadacimab 200 mg (n = 39)*	*Placebo (n = 25)*	
Patienten mit ≥ 1 TEAE	25 (64)	15 (60)	133 (84)^*^
TEAE im Zusammenhang mit der Studienbehandlung	4 (10)	3 (12)	21 (13)
TEAE mit Todesfolge	0	0	0
TEAE, die zum Studienabbruch führten	0	0	1 (1)^‡^
TEAE nach Schweregrad			
Mild	NS	NS	101 (63)
Moderat	NS	NS	81 (50)
Schwer	NS	NS	9 (6)
Schwerwiegende TEAE (SAE)	1 (3)^†^	0	3 (2)^§^
Schwerwiegende TEAE bezogen auf Studienbehandlung	0	0	0
AESI laut Protokoll^¶^	0	0	0
Häufigste TEAE (≥ 5 % der Patienten)			
URTI (Infektion der oberen Atemwege)	4 (10)	2 (8)	9 (6)
Nasopharyngitis	3 (8)	1 (4)	27 (17)
Kopfschmerzen	3 (8)	4 (16)	10 (6)
ISR^**^ (Reaktion an der Injektionsstelle)	2 (5)	3 (12)^††^	19 (12)
COVID‐19	–	–	58 (36)
Influenza	–	–	11 (7)

*Abk*.: AESI, unerwünschtes Ereignis von besonderem Interesse; HAE‐C1INH, hereditäres Angioödem mit C1‐Esterase‐Inhibitor‐Mangel oder ‐Dysfunktion; ISR, Reaktion an der Injektionsstelle; NS (n. a.), nicht angegeben; OLE, offene Erweiterungsstudie; SAE, schwerwiegendes unerwünschtes Ereignis; TEAE, therapiebedingtes unerwünschtes Ereignis; URTI, Infektion der oberen Atemwege.

Daten sind als Anzahl der Patienten mit Ereignissen angegeben, dargestellt als n (%).

* Primäre Endpunkte bewertet basierend auf dem Sicherheitskollektiv der Primäranalyse von Patienten mit HAE‐C1INH (n = 159);

^†^ Ein schweres SAE (Larynxattacke) wurde als nicht mit der Studienbehandlung zusammenhängend bewertet: der Patient erholte sich vollständig und verblieb eine Nacht zur Beobachtung im Krankenhaus

^‡^ Ein Garadacimab‐bedingtes TEAE, das nach 4 Behandlungsmonaten auftrat (Reizung an der Injektionsstelle am Abdomen von moderater Schwere, innerhalb von 24 h nach Injektion; abgeklungen nach 13 Tagen)

^§^ Drei SAEs wurden in der OLE berichtet (COVID‐19, n = 2; abdominale HAE‐Attacke, n = 1); keines stand im Zusammenhang mit Garadacimab

^¶^ AESIs (unerwünschte Ereignisse von besonderem Interesse) laut Protokoll umfassen abnorme Blutungsereignisse, thromboembolische Ereignisse, schwere Überempfindlichkeit und Anaphylaxie

** In der VANGUARD‐Studie waren alle ISR mild; in der OLE‐Studie waren die meisten ISR‐Ereignisse (43) mild (41, 95 %) und die verbleibenden zwei von moderater Schwere

^††^ Eine Reaktion an einer Impfstelle wurde als ISR erfasst.

In der Phase‐III‐OLE‐Studie entsprach das Sicherheitsprofil von Garadacimab im Allgemeinen dem der zulassungsrelevanten Phase‐III‐Studie (VANGUARD). In der gesamten OLE‐Studienpopulation trat bei 135/161 (84 %) der Patienten mindestens ein TEAE auf, wobei bei 21/161 (13 %) der Patienten mindestens ein TEAE im Zusammenhang mit Garadacimab stand (Tabelle 4).^33^

Über das gesamte klinische Entwicklungsprogramm hinweg wurden keine Todesfälle gemeldet.^28^, ^32^, ^33^, ^43^ Ein TEAE führte zum Studienabbruch (eine moderate, Garadacimab‐assoziierte Reaktion an der Einstichstelle [ISR], die nach Monat 4 in der Phase‐III‐OLE‐Studie auftrat).^33^

Die von mit Garadacimab behandelten Patienten erfahrenen TEAE waren überwiegend leichter oder moderater Natur.^28^, ^31^, ^33^ Während des klinischen Entwicklungsprogramms von Garadacimab wurde eine begrenzte Anzahl an SAE gemeldet: In der zulassungsrelevanten Phase‐III‐Studie (VANGUARD) erlitt ein Patient im Garadacimab‐Arm eine Larynxattacke, die eine stationäre Überwachung über Nacht erforderte, von der sich der Patient jedoch vollständig erholte. In der Phase‐III‐OLE‐Studie wurden drei SAE gemeldet (COVID‐19, n = 2; abdominale HAE‐Attacke, n = 1). Keines der SAE wurde von den Prüfärzten als im Zusammenhang mit Garadacimab stehend erachtet.^32^, ^33^

Es traten keine behandlungsbedingten AESI gemäß Protokoll (abnorme Blutungen, thromboembolische Ereignisse, schwere Überempfindlichkeit und Anaphylaxie) in der 6‐monatigen Phase‐III‐Studie (VANGUARD), der Phase‐III‐OLE‐Studie (mediane Exposition 13,8 Monate) oder im TP2 der Phase‐II‐Studie (circa 1,7 Jahre Exposition) auf.^31^, ^32^, ^33^ Während TP2 der Phase‐II‐Studie wurde ein Fall von leichter Epistaxis bei einem mit monatlich 600 mg Garadacimab behandelten Patienten beobachtet; dies stand nicht im Zusammenhang mit Garadacimab und erforderte keine Behandlung.^31^

Die am häufigsten berichteten TEAE (≥ 5 % der Patienten) in der VANGUARD‐Studie umfassten Infektionen der oberen Atemwege (*upper respiratory tract infections*; URTI), Nasopharyngitis, Kopfschmerzen und *injection site reactions* (ISR); in der Phase‐III‐OLE‐Studie waren die am häufigsten gemeldeten TEAE COVID‐19, Nasopharyngitis, ISR, Influenza, Kopfschmerzen und URTI.^32^, ^33^

In einem Pharmakokinetik‐Modell basierend auf gepoolten Daten der Phasen I, II und III wurden keine medikamentösen Wechselwirkungen bei Patienten festgestellt, die neben Garadacimab gleichzeitig Analgetika, Antihistaminika, Antiphlogistika oder Antibiotika erhielten. Konsistent mit den Gesamtstudienpopulationen standen die meisten (91,9 %) TEAE nicht im Zusammenhang mit der Garadacimab‐Behandlung, und 97,6 % waren von leichtem oder moderatem Schweregrad. Alle Garadacimab‐bedingten TEAE waren leicht oder moderat; ISR und Kopfschmerzen waren die am häufigsten berichteten Ereignisse.^46^ Es wurden keine abnormen Blutungsereignisse bei Patienten gemeldet, die Garadacimab zusammen mit einer Begleittherapie mit Antikoagulanzien oder Thrombozytenaggregationshemmern erhielten, und Garadacimab hatte über das gesamte klinische HAE‐Programm hinweg keinen Einfluss auf die Hämostase.^47^

### Einfluss von Garadacimab auf die Lebensqualität (HRQoL) und patientenberichtete Ergebnisse

In der VANGUARD‐Studie wurden klinisch bedeutsame Verbesserungen (≥ 6 Punkte) im Gesamtscore des Lebensqualitätsfragebogens für Angioödeme (AE‐QoL) bereits 31 Tage nach der ersten Gabe von Garadacimab (2 × 200 mg Initialdosis) beobachtet, mit einer Reduktion von 23,7 Punkten gegenüber dem *Run‐in*‐Zeitraum. Diese Verbesserung hielt bis Tag 182 mit einer Reduktion von 26,5 Punkten an. Bei Patienten unter Placebo wurde zu keinem Zeitpunkt der Studie eine Verbesserung des AE‐QoL‐Gesamtscores von ≥ 6 Punkten erreicht.^32^

Die mit Garadacimab assoziierten Verbesserungen des AE‐QoL‐Scores hielten in der Phase‐III‐OLE‐Studie an, in der 82 % der auswertbaren, zuvor mit Garadacimab behandelten Patienten entweder stabil blieben oder weitere klinisch bedeutsame Verbesserungen gegenüber dem AE‐QoL‐Endwert der Vorstudie aufwiesen. Unterdessen zeigten 92 % der auswertbaren Patienten, die zuvor nicht mit Garadacimab behandelt worden waren (Garadacimab‐naiv), klinisch bedeutsame Verbesserungen des AE‐QoL‐Gesamtscores gegenüber dem Ausgangswert.^48^ Sowohl in der VANGUARD‐ als auch in der OLE‐Studie wiesen Patienten, die attackenfrei blieben, die größten Verbesserungen im AE‐QoL‐Score und die höchste Behandlungszufriedenheit auf.^49^

Darüber hinaus bewertete in der VANGUARD‐Studie ein höherer Anteil der mit Garadacimab behandelten Patienten (82 %) das Ansprechen auf die Therapie gemäß dem *Subject's Global Assessment of Response to Therapy* als „gut“ oder „ausgezeichnet“, verglichen mit 33 % in der Placebogruppe.^32^ Dieselbe Entwicklung zeigte sich bis Monat 12 in der OLE‐Studie, in der die Verbesserungen der Patientenbewertungen unter Garadacimab ebenfalls anhielten (93 % bewerteten das Ansprechen als „gut“ oder „ausgezeichnet“).^33^

Verschiedene andere Fragebogen, wie der zur Arbeitsproduktivität und Aktivitätseinschränkung (*Work Productivity and Activity Impairment Questionnaire*, WPAI) und der Fragebogen zur Behandlungszufriedenheit für Medikamente in der Version II (*Treatment Satisfaction Questionnaire for Medication*, TSQM‐II), haben gezeigt, dass Garadacimab die patientenberichteten Outcomes verbessert.^31^, ^48^

### Garadacimab bei jugendlichen und pädiatrischen Patienten

Eine Subgruppenanalyse von Jugendlichen (Alter 12–17 Jahre) der zulassungsrelevanten Phase‐III‐Studie (VANGUARD) und der Phase‐III‐OLE‐Studie (Datenstichtag: 13. Februar 2023; mediane Exposition: 16,3 Monate) zeigte, dass Garadacimab die mittlere monatliche Attackenrate innerhalb dieser Subgruppe gegenüber dem *Run‐in* reduzierte.^50^ In der zulassungsrelevanten Phase‐III‐Studie (VANGUARD) (n = 6) wurde die Reduktion der mittleren monatlichen Attackenrate für vier einzelne mit Garadacimab behandelte Patienten (–5,8 %, 92,3 %, 100 %, 100 %) und zwei mit Placebo behandelte Patienten (–12,2 %, 90,8 %) gegenüber dem *Run‐in* dargestellt. In der OLE‐Studie (n = 10) reichte die Reduktion der mittleren monatlichen Attackenrate gegenüber dem *Run‐in* von 68,2 % bis 100 %, wobei vier Patienten attackenfrei wurden (100 % Reduktion). Garadacimab war bei jugendlichen Patienten gut verträglich, ohne Todesfälle, Therapieabbrüche, SAE, protokollgemäße AESI oder Garadacimab‐bedingte TEAE.^50^

Um die Evidenzbasis für Garadacimab bei HAE über alle Altersgruppen hinweg zu erweitern, wird Garadacimab in einer laufenden pädiatrischen Phase‐III‐Studie evaluiert, deren Abschluss für 2026 erwartet wird; weitere Daten werden zu gegebener Zeit verfügbar sein.^34^

### Garadacimab bei Patienten mit HAE‐nC1INH

Patienten mit HAE‐nC1INH wurden in die Phase‐II‐ und Phase‐III‐OLE‐Studien von Garadacimab eingeschlossen.^51^ Von den sechs Patienten mit bestätigtem HAE aufgrund einer FXII‐Mutation (HAE‐FXII; n = 3) oder aufgrund einer Plasminogen‐Mutation (HAE‐PLG; n = 3), die im Behandlungszeitraum 1 (TP1) der Phase‐II‐Studie offen 600 mg Garadacimab s.c. monatlich erhielten, wechselten zwei Patienten mit HAE‐FXII in TP2 (600 mg Garadacimab s.c.) und später in die Phase‐III‐OLE über, in der sie 200 mg Garadacimab s.c. erhielten. Die anderen vier Patienten brachen die Studie während (n = 1) oder nach TP1 (n = 3) unter Berufung auf mangelnde oder inkonsistente Wirksamkeit ab. Zwei Patienten mit HAE‐FXII erfuhren in der Phase‐II‐Studie eine Reduktion der mittleren monatlichen Attackenrate von > 88 % gegenüber dem *Run‐in* und waren in der Phase‐III‐OLE attackenfrei, während der dritte Patient mit HAE‐FXII eine 19%ige Abnahme der mittleren monatlichen HAE‐Attackenrate aufwies. Während der Phase‐II‐Studie erfuhren zwei Patienten mit HAE‐PLG einen Anstieg der mittleren monatlichen Attackenrate (auf 198 % beziehungsweise 119 %), und der dritte Patient mit HAE‐PLG wies eine 45%ige Abnahme der monatlichen Attackenrate auf. Garadacimab war bei allen sechs Patienten mit HAE‐nC1INH gut verträglich. In der Phase‐II‐Studie traten bei vier der sechs Patienten (HAE‐FXII, n = 2; HAE‐PLG, n = 2) während TP1 TEAE auf, darunter eine leichte Garadacimab‐bedingte ISR und ein nicht im Zusammenhang stehendes SAE einer schweren HAE‐Attacke. Die zwei Patienten mit HAE‐FXII, welche die Studie fortsetzten, erfuhren 13 leichte/moderate TEAE während TP2 der Phase‐II‐Studie und zwei leichte TEAE während der Phase‐III‐OLE.^51^

Es ist zu beachten, dass nur begrenzte Daten zur Anwendung von Garadacimab bei Patienten mit HAE‐nC1INH vorliegen. Patienten mit HAE‐nC1INH stellen eine heterogene Population mit hohem ungedecktem Therapiebedarf dar. Aktuelle Behandlungsstrategien für Patienten mit HAE‐nC1INH basieren auf Evidenz, die primär bei Patienten mit HAE‐C1INH generiert wurde.^51^ Darüber hinaus sprechen einige Subkategorien von HAE‐nC1INH möglicherweise nicht auf eine Behandlung mit Garadacimab an, da die Bradykininproduktion über einen alternativen, FXII‐unabhängigen Weg erfolgt. Es wird empfohlen, eine genetische Testung gemäß den aktuellen HAE‐Leitlinien durchzuführen, sofern verfügbar, und die Behandlung mit Garadacimab abzubrechen, wenn innerhalb von 3 Monaten kein klinisches Ansprechen beobachtet wird.^25^

## GARADACIMAB IN DER AKTUELLEN THERAPIELANDSCHAFT

Das oberste Ziel der HAE‐Behandlung gemäß der WAO/EAACI‐Leitlinie ist das Erreichen einer vollständigen Krankheitskontrolle und die Normalisierung des Alltags der Patienten.^2^ Die Normalisierung des Alltags umfasst sowohl die Reduktion der Attackenrate (wie in der WAO/EAACI‐Leitlinie festgelegt) als auch die Linderung der Krankheitslast.^2^, ^3^, ^6^, ^52^, ^53^

Eine beträchtliche Untergruppe von Patienten mit HAE‐C1INH erfährt unter den derzeit verfügbaren prophylaktischen Strategien weiterhin eine unzureichende Krankheitskontrolle;^54^, ^58^, ^59^ daher werden neuartige LTP‐Behandlungsoptionen benötigt, um den Patienten zu helfen, dem Ziel einer vollständigen Krankheitskontrolle näher zu kommen.

Garadacimab weist einen Wirkmechanismus auf, der sich von den anderen bestehenden LTP‐Behandlungen unterscheidet. Es hemmt FXIIa des Kallikrein‐Kinin‐Systems, was laut einem aktuellen mechanistischen Modell ein effektiverer Behandlungsansatz zur Prävention der Bradykininbildung ist als die Hemmung nachgeschalteter (*downstream*) Enzyme.^27^, ^60^ Frühe Studien an Mausmodellen zeigten zudem, dass 3F7, der Vorläufer‐Antikörper von Garadacimab, eine stärkere Hemmung der Bradykininbildung aufweist als andere nachgeschaltete Inhibitoren, wie zum Beispiel Bradykinin‐B2‐Rezeptor‐Inhibitoren und aktive Plasma‐Kallikrein‐Blocker.^27^, ^29^ Das therapeutische Potenzial von Garadacimab in der aktuellen HAE‐Landschaft ist für Ärzte und Patienten gleichermaßen vielversprechend. Bisher hat das klinische Programm gezeigt, dass Garadacimab eine dauerhafte Wirksamkeit mit einem frühen Schutzbeginn vor HAE‐Attacken besitzt, die über etwa 2 Jahre in den klinischen Phase‐II‐ und Phase‐III‐Studien anhielt. Dies bietet den Patienten das Potenzial, über längere Zeiträume beschwerdefrei zu leben.^28^, ^31^, ^32^, ^33^, ^43^, ^44^ Darüber hinaus war ein hoher Anteil der mit Garadacimab behandelten Patienten während der gesamten zulassungsrelevanten Phase‐III‐ und OLE‐Studien attackenfrei, was das Potenzial verdeutlicht, Patienten näher an das Ziel einer vollständigen Krankheitskontrolle gemäß der WAO/EAACI‐Leitlinie zu bringen.^32^, ^33^, ^49^ Zusätzlich übersetzte sich die mit Garadacimab gezeigte Wirksamkeit innerhalb eines Monats nach Behandlungsbeginn in eine anhaltende und klinisch bedeutsame Verbesserung der gesundheitsbezogenen Lebensqualität (HRQoL), was eine Normalisierung des Alltags bei einer großen Anzahl von Patienten erleichterte, insbesondere bei jenen, die attackenfrei blieben.^32^, ^48^, ^49^ Garadacimab hat zudem ein günstiges Langzeit‐Sicherheitsprofil mit überwiegend leichten/moderaten TEAEs, niedrigen Raten an ISR und keinen protokollgemäßen AESI.^31^, ^32^, ^33^ Diese Sicherheits‐ und Wirksamkeitsdaten sind in der jugendlichen Subgruppenpopulation konsistent, und weitere Untersuchungen in pädiatrischen Populationen werden erwartet, welche die Daten zu Garadacimab über alle Altersgruppen hinweg erweitern werden.^34^, ^50^

Unkontrolliertes HAE macht oft Besuche in der Notaufnahme erforderlich, und Patienten mit unzureichender Krankheitskontrolle leiden unter starker Angst und Depression in Erwartung ihrer nächsten Attacke.^3^, ^54^ Derzeit verfügbare Therapien erfordern eine häufige Dosierung von bis zu zweimal wöchentlich, und HAE‐Patienten müssen unter Umständen ihren Tagesablauf an ihr Dosierungsschema anpassen.^2^, ^61^, ^62^ In einer US‐Umfrage aus dem Jahr 2020 berichteten 20 % der Patienten, dass die Zeit für die Verabreichung der prophylaktischen Behandlung ihr tägliches Leben negativ beeinflusste, wobei einige angaben, Medikamente aufgrund von Unannehmlichkeiten bei der Behandlung ausgelassen zu haben.^2^ Zusätzlich können eine verbesserte Wirksamkeit der LTP, bessere Verträglichkeit sowie optimierte Verabreichungswege und ‐schemata die HRQoL der Patienten positiv beeinflussen. Neuartige LTP‐Therapien können eine komfortable Dosierung bieten, um Adhärenz und HRQoL der Patienten zu verbessern.^55^, ^56^, ^57^, ^61^, ^62^ Neben seinem Wirksamkeits‐ und Sicherheitsprofil hat Garadacimab 200 mg s.c. einmal monatlich das Potenzial, den Komfort der Verabreichung, die Patientencompliance und die HRQoL aufgrund seines Dosierungsschemas und der Art der Anwendung zu verbessern und so die Beeinträchtigung des täglichen Lebens der Patienten zu minimieren.^28^, ^32^, ^33^, ^48^

## ZUKUNFTSPERSPEKTIVEN

Basierend auf der bisherigen klinischen Evidenz zeigt Garadacimab Potenzial als neue Behandlungsoption in der HAE‐Landschaft. Es bietet Patienten die Möglichkeit, den in aktuellen klinischen Leitlinien festgelegten Behandlungszielen einer vollständigen Krankheitskontrolle und Normalisierung des Lebens so nahe wie möglich zu kommen. Langzeitdaten zur Sicherheit und Wirksamkeit werden weiterhin in der laufenden Phase‐III‐OLE‐Studie erhoben, und die Eignung von Garadacimab für pädiatrische Patienten wird derzeit evaluiert.^33^, ^34^ Dies ist besonders wichtig und stellt einen erheblichen ungedeckten therapeutischen Bedarf dar, da pädiatrische Patienten nur begrenzte Optionen für eine Langzeitprophylaxe (LTP) haben: Bisher ist lediglich Lanadelumab für Patienten ab 2 Jahren und pdC1INH für Patienten ab 6 Jahren zugelassen.^6^, ^18^, ^19^, ^20^, ^22^

## SCHLUSSFOLGERUNGEN

Garadacimab, subkutan verabreicht (Initialdosis von 2 × 200 mg, gefolgt von 200 mg einmal monatlich), ist ein neuartiger, hochaffiner, vollhumaner rekombinanter monoklonaler Immunglobulin‐G4‐Antikörper, der gezielt gegen FXIIa im Kallikrein‐Kinin‐System gerichtet ist. Garadacimab ist in mehreren Ländern als LTP zur routinemäßigen Prävention von HAE‐Attacken zugelassen. Darüber hinaus zeigte Garadacimab über das gesamte klinische Programm hinweg einen frühen Wirkungseintritt sowie eine dauerhafte Wirksamkeit mit anhaltendem Schutz vor HAE‐Attacken bei einem günstigen Langzeit‐Sicherheitsprofil. Garadacimab könnte potenziell dazu beitragen, Patienten mit HAE dem Erreichen der leitliniengemäßen Ziele von Krankheitskontrolle und Normalisierung des Lebens näherzubringen.

## DANKSAGUNG

Die Unterstützung bei der Erstellung des Manuskripts (*Medical Writing*) erfolgte unter der Leitung der Autoren durch Arezou Seyed Hossein, MPharm, von Helix, OPEN Health Communications, London, GB, und wurde von CSL Behring, LLC, in Übereinstimmung mit den GPP‐2022‐Leitlinien (*Good Publication Practice*) finanziert.

## FINANZIERUNG

Die Finanzierung für die Unterstützung bei der Erstellung des Manuskripts (*Medical Writing*) wurde von CSL Behring, LLC, bereitgestellt.

## INTERESSENKONFLIKT

E.A.‐P. hat persönliche und/oder institutionelle Honorare als Referent/Berater erhalten und/oder wurde durch Forschungsförderung/Unterstützung als Prüfarzt für klinische Studien von Astria Therapeutics, BioCryst, BioMarin, CSL Behring, Intellia Therapeutics, KalVista, Otsuka, Pharming, Pharvaris und Takeda/Shire außerhalb der eingereichten Arbeit unterstützt. R.T. erhielt persönliche Honorare von Takeda und CSL Behring und nahm an klinischen Studien teil, die von Takeda und CSL Behring außerhalb der eingereichten Arbeit initiiert wurden. P.S. erhielt Honorare, Forschungsförderung und Reisekostenzuschüsse von und/oder war als Berater tätig für und/oder nahm an Advisory Boards teil für BioCryst, CSL Behring, KalVista, Octapharma, Pharming, Pharvaris, Shire und Takeda außerhalb der eingereichten Arbeit. I.M.S. erhielt Unterstützung durch Forschungsförderung und/oder Referenten‐/Beraterhonorare von BioCryst, CSL Behring, KalVista Pharmaceuticals, Octapharma, Pharming, Pharvaris und Takeda/Shire außerhalb der eingereichten Arbeit. T.L. ist Angestellter der CSL Behring GmbH. M.M. hat Honorare als Referent/Berater von Astria, BioCryst, CSL Behring, Intellia, KalVista, Octapharma, Otsuka, Pharvaris und Takeda/Shire außerhalb der eingereichten Arbeit erhalten.
